# Perceived addiction to smoking and associations with motivation to stop, quit attempts and quitting success: A prospective study of English smokers

**DOI:** 10.1016/j.addbeh.2018.11.030

**Published:** 2019-03

**Authors:** Olga Perski, Natalie Herd, Robert West, Jamie Brown

**Affiliations:** a Department of Clinical, Educational and Health Psychology, University College London, 1-19 Torrington Place, London WC1E 6BT, UK; b Department of Behavioural Science and Health, University College London, 1-19 Torrington Place, London WC1E 6BT, UK

**Keywords:** Smoking, Tobacco, Quit attempts, Perceived addiction, Disease model of addiction, Motivation to stop

## Abstract

**Aims:**

Some argue that perceived addiction to smoking (PAS) might undermine motivation to stop. We examined the association of PAS with motivation to stop in a population sample and assessed its association with past and future quit attempts and future quit success.

**Method:**

12,700 smokers in England were surveyed between September 2009–March 2012 as part of the Smoking Toolkit Study. 2796 smokers were followed up after 6 months. PAS was assessed at baseline by a single self-report item. The outcome variables were ratings of motivation to stop and reports of past-year quit attempts at baseline, and quit attempts in the past 6 months and smoking status at follow-up. Baseline covariates were sex, age, social grade and daily cigarette consumption.

**Results:**

In adjusted analyses, PAS was positively associated with at least some degree of motivation to stop versus no motivation (ORs = 1.97–2.96, all *p*'s < 0.001). PAS was also positively associated with past-year quit attempts (OR = 1.43, 95% CI = 1.32–1.55, *p* < 0.001), but not with future quit attempts (OR = 1.17, 95% CI = 0.99–1.39, *p* = 0.064) or quit success (OR = 1.04, 95% CI = 0.73–1.47, *p* = 0.83).

**Conclusion:**

In smokers in England, perceived addiction to smoking is positively associated with motivation to stop and having recently made a quit attempt but is not clearly associated with future quit attempts or success. These findings provide no grounds for believing that increasing smokers' perceived addiction through promotion of stop-smoking support has undermined motivation to stop.

## Highlights

• Perceived addiction to smoking is positively associated with current motivation to stop.
• Perceived addiction to smoking does not appear to undermine future quit attempts or success.
• The UK emphasis on providing stop-smoking support does not appear to undermine motivation to stop.

## 1 Introduction

Cigarette addiction is an important reason why many people continue to smoke, despite most wanting to quit (Benowitz, 2010; West, 2009). This has led to public health messages encouraging smokers to use pharmacological and behavioural support to aid their quit attempts (Department of Health, 2017). Some research suggests that the belief that one is addicted to smoking (‘perceived addiction to smoking’ – PAS) negatively influences quitting self-efficacy (Eiser, Sutton, & Wober, 1978; Eiser, van der Pligt, Raw, & Sutton, 1985), which some argue may deter smokers from making a quit attempt (Heather et al., 2017). If so, public health messages highlighting the addictive nature of smoking and the benefits of using stop-smoking aids may be counterproductive by reducing quit attempt rates. This study aimed to test this hypothesis by examining the association of PAS with current motivation to stop smoking in a population sample. It also examined the association of PAS with recent quit attempts, future quit attempts and future quit success.

Viewed from a theoretical perspective, endorsing the belief that one is addicted to smoking is thought to be synonymous with the belief that one is continuing to smoke despite wanting to quit (Eiser et al., 1985; Wigginton, Morphett, & Gartner, 2017). The Brain Disease Model of Addiction (BDMA) asserts that addiction is a “…chronic, relapsing disease that changes the structure and function of the brain…” (Animal farm, 2014), and is characterised by compulsivity and loss of self-control (Leshner, 1997; Volkow, Koob, & McLellan, 2016). Although its proponents claim that the BDMA can help reduce stigma associated with addiction and encourage treatment-seeking, others argue that neurobiological views of addiction (and by extension PAS) may undermine individuals' motivation to quit (Barnett, Dilkes-Frayne, Savic, & Carter, 2018; Heather et al., 2017).

In smokers, the associations of PAS with motivation to stop, future quit attempts and quitting success are unclear. Beliefs about PAS can develop with only modest tobacco exposure in adolescents, and such beliefs predict self-reported vulnerability to smoking (Okoli, Richardson, Ratner, & Johnson, 2009). PAS has in some research been found to be positively associated with perceived quitting difficulty (Twyman, Bonevski, Paul, & Bryant, 2014) and reduced chances of success amongst those attempting to quit (Chaiton et al., 2017; Eiser et al., 1978). However, in other studies, the perceived likelihood of being able to quit appears unrelated to PAS (Martin, 1990; Morphett et al., 2017). It has been also found that PAS is associated with a greater desire to stop smoking (Eiser et al., 1978; Pechacek et al., 2017) and increased chances of making a quit attempt (Chaiton et al., 2017).

If PAS undermines motivation to stop and reduces the probability of trying to quit, this has important policy implications. Countries such as the United Kingdom (UK), which have focused tobacco control efforts on providing support for smokers wanting to quit, may have inadvertently reduced smokers' chances of making a quit attempt. If this effect is large enough, it could lead to fewer smokers quitting than if no support programme were offered.

The Smoking Toolkit Study (STS) is an ongoing series of monthly national surveys in England. Between 2009 and 2012 it assessed PAS as well as motivation to stop smoking, quit attempts and quit success amongst those making an attempt (Fidler et al., 2011). It therefore provided an opportunity to test the hypothesis that higher PAS is associated with reduced motivation to stop at an individual level. As the STS also included a 6-month follow-up survey, it was also able to assess whether PAS was associated with future quit attempts and quit success. The STS also collects data on age, sex, daily cigarette consumption and social grade (an occupation-based measure of socio-economic status).

The research questions addressed by the present study were:

1. Do smokers who endorse the belief that they are addicted to smoking have reduced motivation to stop smoking, with and without adjustment for age, sex, daily cigarette consumption and social grade?
2. Do smokers who endorse the belief that they are addicted to smoking have a greater probability of a recent past quit attempt, future quit attempts and quit success given an incident attempt, with and without adjustment for age, sex, daily cigarette consumption and social grade?

## 2 Method

### 2.1 Study design and setting

The STROBE guidelines were used in the design and reporting of this study (Von Elm et al., 2007). This was a correlational study involving cross-sectional and prospective survey data.

The study was part of the ongoing Smoking Toolkit Study, which involves monthly face-to-face, computer-assisted household surveys in England and has been in operation since 2006 (Fidler, Shahab, West, Jarvis, et al., 2011). The sample is a hybrid of a random probability and quota sample, which has been shown to result in a sample that is representative of the adult population of smokers in England (Fidler, Shahab, West, Jarvis, et al., 2011). Interviews were held with one member of each household. Informed consent was obtained prior to each interview. All smokers who agreed to be re-contacted were sent postal follow-up questionnaires 6 months after the baseline assessment. One reminder letter was sent. Ethical approval was provided by UCL's Research Ethics Committee (0498/001).

### 2.2 Study population

Data included in the present study were collected from respondents surveyed between September 2009 and March 2012 (when PAS was included in the survey), with the exception of May 2010 (a wave in which PAS was not measured). Respondents were included in the analyses if they smoked cigarettes (manufactured or hand-rolled) or any other tobacco product (e.g. pipe, cigar) at least weekly at the time of the baseline survey and were aged 16 years or over.

### 2.3 Measures

The independent variable was PAS measured at baseline. This variable was derived from one of two items depending on the survey wave (i.e. “Which of the following apply to you?”; “How do you feel about smoking?”), both of which asked participants to either select or not select the response option: “I am addicted to smoking/cigarettes”. Responses were coded 0 for those who did not select this option and 1 for those who did select this option. A single self-report measure of PAS has previously been used to capture this construct (Chaiton et al., 2017; Eiser et al., 1985; Pechacek et al., 2017; Sendzik, McDonald, Brown, Hammond, & Ferrence, 2011).

The dependent variables were:

*Motivation to stop smoking.* This was measured at baseline by the Motivation to Stop Scale (MTSS; Kotz, Brown, & West, 2013), which asks: “Which of the following best describes you?” The response options were: 1) “I don't want to stop smoking”; 2) “I think I should stop smoking but don't really want to”; 3) “I want to stop smoking but haven't thought about when”; 4) “I REALLY want to stop smoking but I don't know when I will”; 5) “I want to stop smoking and hope to soon”; 6) “I REALLY want to stop smoking and intend to in the next 3 months”; 7) “I REALLY want to stop smoking and intend to in the next month”. An *a priori* decision to dichotomise responses into high (6–7) and low (1–5) motivation was made to aid interpretation (reported in Supplementary File 1), but the analysis plan was amended as a result of the review process to one in which each motivation to stop category was compared with the lowest category.

*Recent quit attempts.* This was measured at baseline by asking: “How many serious attempts to stop smoking have you made in the past 12 months? By serious I mean you decided that you would try to make sure you never smoked again.” This item was coded 0 for smokers who responded that they had not made a quit attempt, and 1 for one or more quit attempts.

*Future quit attempts.* This was measured at 6-month follow-up by two items: a) “Have you made a serious attempt to stop smoking in the past 12 months?”; b) “How long ago did your most recent quit attempt start?”. Responses were coded 1 for those who responded that they had made a quit attempt that started less than 6 months ago and 0 otherwise.

*Future quit success.* This was measured at 6-month follow-up by asking those who had made a quit attempt in the past 6 months: “How long did your most recent quit attempt last before you went back to smoking?”. Responses were coded 1 for those who reported that they were still not smoking and 0 otherwise.

Respondents also provided data at baseline on age, sex, social grade (AB = managerial and professional occupations, C1 = intermediate occupations, C2 = small employers and own account workers, D = lower supervisory and technical occupations, and E = semi-routine and routine occupations, never workers, and long-term unemployed) and daily cigarette consumption (converted to daily consumption for non-daily smokers who reported the number of cigarettes smoked per week) (Fidler, Shahab, West, Jarvis, et al., 2011).

### 2.4 Data analysis

Data were analysed using SPSS version 21.0 (IBM Corp., 2012). The analysis plan was pre-registered on the Open Science Framework (https://doi.org/10.17605/OSF.IO/3VWZS). Differences between the follow-up sample and those not followed up were assessed by χ^2^ tests and independent samples *t*-tests for categorical and continuous variables, respectively. Participants with missing data for any of the variables in the analyses were excluded.

The association of PAS with motivation to stop was assessed by logistic regression of dichotomised motivation to stop on PAS, adjusting and not adjusting for all covariates (see Supplementary File 1). Following the review process, we undertook a multinomial logistic regression of the lowest category of motivation to stop against all higher categories, adjusting and not adjusting for all covariates. The associations of PAS with recent quit attempts, future quit attempts and future quit success were assessed by logistic regression of these dependent variables on PAS, adjusting and not adjusting for all covariates.

Unplanned further analyses were undertaken to examine whether non-significant associations could best be characterised as evidence of no effect or whether data were insensitive (Depaoli, Rus, Clifton, van de Schoot, & Tiemensma, 2017; Dienes, 2011, Dienes, 2016). Bayes Factors (BF), with the alternative hypotheses conservatively represented by half-normal distributions, were calculated using an online calculator (http://www.lifesci.sussex.ac.uk/home/Zoltan_Dienes/inference/Bayes.htm). In an alternative hypothesis represented by a half-normal distribution, the standard deviation of a distribution can be specified as an expected effect size, meaning that plausible values are effectively represented between zero and twice the effect size, with smaller values being more likely. The expected effect sizes were set to be the same as the logarithm of the lower bounds of the 95% CIs of the effect sizes observed in the study by Chaiton and colleagues (i.e. OR = 1.88 and OR = 2.01, respectively) (Chaiton et al., 2017). BFs were also calculated for the logarithm of the point estimates of the effects (i.e. OR = 2.49 and OR = 2.93, respectively). A BF ≥ 3 can be interpreted as substantial evidence for the alternative hypothesis (and against the null), while a BF of ≤ 1/3 can be interpreted as evidence for the null hypothesis. BFs between 1/3 and 3 suggest that the data are insensitive to distinguish the alternative hypothesis from the null (Dienes, 2011).

We also conducted an unplanned sensitivity analysis repeating the analysis of the prospective association of PAS with future quit attempts, adjusting for motivation to stop. This was to test the hypothesis that PAS may undermine quit attempts independently of any effect on motivation to stop.

## 3 Results

A total of 12,970 smokers were surveyed between September 2009 and March 2012, of whom 12,700 (97.9%) provided complete data on all baseline variables. Participant characteristics are reported in Table 1. A total of 2796 participants (21.6%) responded to the 6-month follow-up questionnaire. Those who responded at follow-up were more likely than non-responders to: be female (χ^2^(1) = 42.3, *p* < 0.001), be older (χ^2^(5) = 369.6, *p* < 0.001), be from a higher social grade (χ^2^(4) = 13.1, *p* < 0.05), report feeling addicted to smoking (χ^2^(1) = 41.2, *p* < 0.001), have lower motivation to stop smoking (χ^2^(6) = 41.2, *p* < 0.001) and smoke more cigarettes per day (*t*(12,698) = −7.026, *p* < 0.001).

**Table 1:** Participant demographic and smoking characteristics.

	Baseline sample (*N* = 12,700)	Follow-up sample (*N* = 2796)
Demographic characteristics		
Sex, % (N)		
Female	50.1 (6369)	55.6 (1554)
Male	49.9 (6331)	44.4 (1242)
Age, % (N)		
16–24	17.3 (2191)	9.1 (254)
25–34	20.9 (2648)	15.1 (423)
35–44	19.7 (2499)	19.5 (545)
45–54	16.9 (2150)	21.5 (600)
55–64	13.4 (1706)	19.1 (534)
65+	11.9 (1506)	15.7 (440)
Social grade, % (N)		
AB	9.8 (1241)	10.6 (297)
C1	21.4 (2717)	20.0 (559)
C2	21.4 (2716)	21.6 (605)
D	19.1 (2423)	17.7 (495)
E	28.4 (3603)	30.0 (840)

Smoking characteristics		
Perceived addiction to smoking, % (N)		
No	63.7 (8090)	58.5 (1637)
Yes	36.3 (4610)	41.5 (1159)
Motivation to stop smoking, % (N)		
1) “I don't want to stop smoking”	20.7 (2623)	21.0 (586)
2) “I think I should stop smoking but don't really want to”	12.7 (1615)	15.8 (442)
3) “I want to stop smoking but haven't thought about when”	9.5 (1201)	8.7 (243)
4) “I REALLY want to stop smoking but I don't know when I will”	23.2 (2946)	23.7 (662)
5) “I want to stop smoking and hope to soon”	12.1 (1543)	11.1 (310)
6) “I REALLY want to stop smoking and intend to in <3 months”	11.7 (1487)	11.1 (309)
7) “I REALLY want to stop smoking and intend to in <1 month”	10.1 (1285)	8.7 (244)
Past quit attempts, % (N)		
No	68.5 (8704)	69.9 (1954)
Yes	31.5 (3996)	30.1 (842)
Prospective quit attempts, % (N)		
No	N/A	70.0 (1957)
Yes	N/A	30.0 (839)
Cigarettes smoked per day, mean (*SD*)	13.0 (*8.5*)	14.0 (*8.7*)

Subgroup attempting to quit at follow-up (*N* = 839)		
Quitting success, % (N)		
No	N/A	77.7 (652)
Yes	N/A	22.3 (187)

Table 2 shows the results of the unadjusted and adjusted multinomial logistic regression of motivation to stop smoking on PAS. Compared with the lowest category of motivation, all levels of motivation were higher in respondents who reported that they felt addicted to smoking (ORs_adj_ = 1.97–2.96, all *p*'s < 0.001).

**Table 2:** Unadjusted and adjusted odds ratios (ORs) for the association between PAS and each level of motivation to stop smoking, compared with no motivation (“I don't want to stop smoking”).

	“I think I should stop smoking but don't really want to”		“I want to stop smoking but haven't thought about when”		“I REALLY want to stop smoking but I don't know when I will”		“I want to stop smoking and hope to soon”		“I REALLY want to stop smoking and intend to in <3 months”		“I REALLY want to stop smoking and intend to in <1 month”	
	OR (95% CI)	*p*	OR (95% CI)	*p*	OR (95% CI)	*p*	OR (95% CI)	*p*	OR (95% CI)	*p*	OR (95% CI)	*p*
Perceived addiction to smoking												
No	1.00	–	1.00	–	1.00	–	1.00	–	1.00	–	1.00	–
Yes	1.88 (1.64–2.15)	<0.001	1.94 (1.68–2.25)	<0.001	2.61 (2.32–2.93)	<0.001	1.66 (1.45–1.91)	<0.001	2.06 (1.80–2.37)	<0.001	1.84 (1.59–2.12)	<0.001
	OR_adj_ (95% CI)	*p*	OR_adj_ (95% CI)	*p*	OR_adj_ (95% CI)	*p*	OR_adj_ (95% CI)	*p*	OR_adj_ (95% CI)	*p*	OR_adj_ (95% CI)	*p*
Perceived addiction to smoking												
No	1.00	–	1.00	–	1.00	–	1.00	–	1.00	–	1.00	–
Yes	1.97 (1.71–2.26)	<0.001	2.30 (1.98–2.69)	<0.001	2.96 (2.62–3.33)	<0.001	2.07 (1.79–2.39)	<0.001	2.47 (2.14–2.85)	<0.001	2.31 (1.99–2.69)	<0.001
Sex (reference: female)	1.36 (1.20–1.55)	<0.001	1.11 (0.96–1.28)	0.15	1.32 (1.18–1.47)	<0.001	1.18 (1.03–1.34)	<0.05	1.24 (1.08–1.41)	<0.01	1.20 (1.05–1.38)	<0.01
Age (reference: 16–24 years)	0.90 (0.86–0.93)	<0.001	0.77 (0.74–0.81)	<0.001	0.82 (0.79–0.85)	<0.001	0.77 (0.74–0.80)	<0.001	0.78 (0.75–0.81)	<0.001	0.77 (0.74–0.81)	<0.001
Social grade (reference: AB)	0.86 (0.82–0.91)	<0.001	0.92 (0.87–0.97)	<0.01	1.00 (0.96–1.04)	0.84	0.92 (0.88–0.97)	<0.01	0.95 (0.90–0.99)	<0.05	0.92 (0.87–0.97)	<0.01
Cigarettes per day	0.99 (0.99–1.00)	<0.05	0.97 (0.96–0.98)	<0.001	0.98 (0.97–0.98)	<0.001	0.96 (0.95–0.97)	<0.001	0.97 (0.96–0.97)	<0.001	0.95 (0.95–0.96)	<0.001

*Note*. OR_adj_ = ORs are adjusted for sex, age, social grade and cigarettes per day.

Table 3 shows the results of the unadjusted and adjusted logistic regressions of recent quit attempts, future quit attempts and future quit success onto PAS. In adjusted analyses, PAS was significantly associated with greater odds of a recent quit attempt (OR_adj_ = 1.43, 95% CI = 1.32–1.55, *p* < 0.001), but not with future quit attempts (OR_adj_ = 1.17, 95% CI = 0.99–1.39, *p* = 0.064), or with future quit success (OR_adj_ = 1.04, 95% CI = 0.73–1.47, *p* = 0.83).

**Table 3:** Unadjusted and adjusted odds ratios (ORs) for the association between PAS and past quit attempts, future quit attempts and future quit success.

	Recent quit attempts (*N* = 12,700)		Future quit attempts (*N* = 2796)		Future quit success (*N* = 839)	
	OR (95% CI)	*p*	OR (95% CI)	*p*	OR (95% CI)	*p*
Perceived addiction to smoking						
No	1.00	–	1.00	–	1.00	–
Yes	1.38 (1.28–1.49)	< 0.001	1.12 (0.95–1.32)	0.41	0.97 (0.70–1.35)	0.85
	OR_adj_ (95% CI)	*p*	OR_adj_ (95% CI)	*p*	OR_adj_ (95% CI)	*p*
Perceived addiction to smoking						
No	1.00	–	1.00	–	1.00	–
Yes	1.43 (1.32–1.55)	< 0.001	1.17 (0.99–1.39)	0.06	1.04 (0.73–1.47)	0.83
Sex						
Female	1.00	–	1.00	–	1.00	–
Male	0.83 (0.77–0.90)	< 0.001	0.98 (0.83–1.16)	0.82	1.13 (0.81–1.59)	0.47
Age						
16–24	1.00	–	1.00	–	1.00	–
25–34	0.87 (0.78–0.98)	0.02	0.95 (0.68–1.34)	0.78	1.46 (0.74–2.90)	0.28
35–44	0.75 (0.66–0.84)	< 0.001	1.03 (0.75–1.43)	0.85	1.04 (0.53–2.03)	0.91
45–54	0.69 (0.60–0.78)	< 0.001	0.89 (0.64–1.23)	0.48	1.25 (0.64–2.46)	0.51
55–64	0.65 (0.56–0.74)	< 0.001	1.15 (0.83–1.60)	0.39	0.90 (0.45–1.78)	0.75
65+	0.45 (0.39–0.53)	< 0.001	0.99 (0.71–1.39)	0.96	1.03 (0.51–2.10)	0.94
Social grade						
AB	1.00	–	1.00	–	1.00	–
C1	0.89 (0.77–1.03)	0.10	0.94 (0.69–1.29)	0.71	1.07 (0.60–1.92)	0.81
C2	0.98 (0.85–1.14)	0.83	1.02 (0.75–1.38)	0.92	0.95 (0.54–1.68)	0.86
D	0.96 (0.82–1.11)	0.54	0.85 (0.62–1.17)	0.32	0.78 (0.42–1.45)	0.42
E	1.01 (0.88–1.16)	0.89	1.16 (0.87–1.55)	0.30	0.43 (0.24–0.77)	< 0.01
Cigarettes per day	0.99 (0.98–1.00)	< 0.01	0.99 (0.98–1.00)	0.02	0.98 (0.96–1.00)	0.06

*Note.* OR_adj_ = ORs are adjusted for sex, age, social grade and cigarettes per day.

Calculation of BFs indicated that the data on future quit attempts and future quit success marginally favoured the null hypothesis compared with modest associations, but that the data were insensitive to detect an effect (BF = 0.67 and BF = 0.48, respectively). The interpretation did not change materially for somewhat larger associations (BF = 0.5 and BF = 0.38, respectively).

In the sensitivity analysis, PAS was not significantly associated with future quit attempts after adjusting for motivation to stop (OR = 1.01, 95% CI = 0.85–1.21, *p* = 0.90).

## 4 Discussion

This study found that reports of PAS were positively associated with motivation to stop smoking. It was also positively associated with recent quit attempts but not with future quit attempts or quit success given an incident attempt. Calculation of Bayes Factors indicated that the data were insensitive to detection of moderately sized associations of PAS with future quit attempts and quit success.

These results do not support the hypothesis that PAS acts to undermine motivation to stop smoking and as a deterrent to trying to quit smoking. Therefore, there appears to be no reason to believe that the UK's emphasis on providing support for smokers to help them quit is undermining population quit attempt rates. The findings regarding PAS and future quit attempts and quit success leave open the question of whether PAS may promote or undermine quitting success, and whether PAS may be a useful marker of actual cigarette addiction. In contrast, there is clear and consistent evidence that the Fagerström Test for Cigarette Dependence (Kozlowski, Porter, Orleans, Pope, & Heatherton, 1994) and ratings of the strength of urges to smoke (Fidler, Shahab, & West, 2011) predict failure of future quit attempts and therefore are valid measures of cigarette addiction.

The unplanned sensitivity analysis assessing the association of PAS with future quit attempts when adjusting for motivation to stop smoking did not provide evidence that the expression of PAS is indicative of two competing psychological processes (i.e. one motivational and one involving beliefs about the perceived feasibility of quitting). After adjusting for motivation to stop smoking – which was positively associated with PAS – we tested whether there was an independent, negative association between PAS and later quit attempts, whereby PAS undermined the perceived feasibility of quitting. This was not the case; the association between PAS and later attempts remained non-significant after adjusting for baseline motivation.

This study has a number of limitations. The survey relied on self-reported quitting data. As unsuccessful quit attempts may not be accurately recalled, it is possible that those who endorse the belief that they are addicted to smoking also have a greater likelihood of recalling past quit attempts (Berg et al., 2010). As the variable ‘future quit success’ also incorporated respondents who had been quit for only a short period of time (i.e. one week), future research may benefit from limiting analyses to those who have been quit for at least 28 days (West & Stapleton, 2008). The findings may not be generalisable to populations outside of England, and due to differential drop-out, the sample followed up was not completely representative of the baseline sample, which may have biased the results. It should also be noted that data for the final study sample were collected between 2009 and 2012. The market for nicotine products has changed since 2012 with the introduction of popular new nicotine products, such as e-cigarettes, and it is possible that beliefs about addiction have evolved. Future research should assess whether the current findings are robust to such changes. Due to the nature of the measure used to capture the phenomenon of interest (i.e. asking respondents about whether or not they felt addicted to smoking), this study may have failed to incorporate smokers who believe that they are specifically addicted to nicotine or any other aspect of tobacco smoking (Pfeffer, Wigginton, Gartner, & Morphett, 2017). Finally, although the use of a dichotomous measure of PAS aided the interpretation of the findings, a continuous measure may have been able to detect greater variability amongst respondents.

It has been suggested that the act of engaging in a quit attempt and observing the outcome of that attempt is likely to alter beliefs about addiction, as it may affect the individual's sense of autonomy (Chaiton et al., 2017) or self-efficacy (Bandura, 1977). Future research should examine the temporal dynamics of PAS and whether it changes as a function of quit attempts, relapse and quit success in real-time using experience sampling methodology (Stone & Shiffman, 1994).

The current study did not examine whether beliefs about PAS differed depending on dual use of tobacco products (e.g. cigarettes and cigarillos). It is possible that dual use may strengthen the belief that one must be addicted to smoking. This merits exploration in future research studies. Future research could also include a wider range of sociodemographic characteristics (e.g. annual income), that may better translate to populations outside of England.

In conclusion, this study found a positive association of PAS with motivation to stop smoking, and of PAS and recent quit attempts. Findings on the association of PAS with future quit attempts and quit success were inconclusive. Based on the present results, there are no grounds for believing that increasing smokers' perceived addiction to smoking through the promotion of stop-smoking support services has undermined motivation to stop smoking.

## Role of funding sources

Cancer Research UK is the main contributor (C1417/A22962), but the UK Department of Health, Pfizer, GlaxoSmithKline and Johnson and Johnson have also all contributed funding to the data collection for the Smoking Toolkit Study. OP is funded by Bupa under its partnership with University College London. NH is funded by the NIHR HPRU in Evaluation of Interventions. RW and JB receive support from CRUK (C1417/A22962). The funders had no final role in the study design; in the collection, analysis and interpretation of data; in the writing of the report; or in the decision to submit the paper for publication. All researchers listed as authors are independent from the funders and all final decisions about the research were taken by the investigators and were unrestricted.

## Contributors

All authors contributed to the design of the study. OP conducted the statistical analysis and wrote the first draft of the manuscript. All authors have contributed to and have approved the final manuscript.

## Conflict of interest

OP and NH report no competing interests to declare. RW undertakes research and consultancy for and receives travel funds and hospitality from manufacturers of smoking cessation medications (Pfizer, GlaxoSmithKline and Johnson and Johnson). JB has received unrestricted research funding from Pfizer.

## Appendix A Supplementary data

Supplementary File 1 Image 1
