# Supplementary material for: Perceived addiction to smoking and associations with motivation to stop, quit attempts and quitting success: A prospective study of English smokers
Source: Addict Behav. 2019 Mar;90:306–11. doi: 10.1016/j.addbeh.2018.11.030 (PMC6334166; doi:10.1016/j.addbeh.2018.11.030)
Supplement: Supplementary file 1 [file mmc1.docx]

**Supplementary File 1**

**Table.** Odds ratios (ORs) for the association between PAS and the dichotomised motivation to stop smoking variable, as pre-specified in the analysis plan.

|  | **High motivation to stop smoking**  **(reference: low motivation)** | | | | | |
| --- | --- | --- | --- | --- | --- | --- |
|  | **OR (95% CI)** | | | ***p*** | **OR_adj_ (95% CI)** | ***p*** |
| **Perceived addiction to smoking** |  | | |  |  |  |
| No | 1.00 | | | - | 1.00 | - |
| Yes | 1.11 (1.02-1.21) | | | .02 | 1.21 (1.11-1.33) | < .001 |
|  |  |  |  |  |  |  |

*Note.* OR_adj_ = ORs are adjusted for sex, age, social grade and cigarettes per day.
